# Can natural forest expansion contribute to Europe's restoration policy agenda? An interdisciplinary assessment

**DOI:** 10.1007/s13280-023-01924-2

**Published:** 2023-09-29

**Authors:** Theresa Frei, Josep Maria Espelta, Elena Górriz-Mifsud, Arndt Hampe, François Lefèvre, Irene Martín-Forés, Georg Winkel

**Affiliations:** 1 European Forest Institute, Platz der Vereinten Nationen 7, 53113 Bonn, Germany; 2 CREAF, E08193 Bellaterra (Cerdanyola del Vallès), 08193 Barcelona, Catalonia Spain; 3 Forest Science and Technology Center of Catalonia (CTFC), Ctra. St. Llorenç de Morunys km.2, 25280 Solsona, Spain; 4 BIOGECO, INRAE, University Bordeaux, Cestas, Bordeaux, France; 5 INRAE, URFM, 228 route de l’aérodrome AgroParc, 84914 Avignon, France; 6 School of Biological Sciences, The University of Adelaide, Adelaide, SA 5005 Australia; 7 Wageningen University, 6708 PB Wageningen, The Netherlands

**Keywords:** EU policy, Forest policy, Forest regrowth, Land abandonment, Natural succession, Passive restoration

## Abstract

Natural forest expansion (NFE), that is, the establishment of secondary forest on non-forested land through natural succession, has substantially contributed to the widespread expansion of forests in Europe over the last few decades. So far, EU policies have largely neglected the potential of NFE for meeting policy objectives on restoration. Synthesising recent interdisciplinary research, this paper assesses the challenges and opportunities of NFE in view of contributing to European forest and ecosystem restoration. Specifically, we discuss the potential for supporting climate change mitigation and adaptation, biodiversity conservation, and forestry and economic use, summarize the current knowledge about societal perceptions and the policymaking on NFE, and make policy recommendations to better use the potential of NFE. We conclude that NFE has the potential to contribute to the European restoration policy agenda if local contexts and possible trade-offs are properly considered.

## Introduction

Europe has historically faced more habitat fragmentation than any other continent. The region has been the first to undergo a turnaround from diminishing to increasing forest area as a consequence of farmland abandonment. Several Western and Central European countries reached the turning point in the so-called ‘forest transition’ in the nineteenth century, others in Southern Europe during the first or second half of the twentieth century (Kauppi et al. 2018). Since 1950, Europe's forests have increased by > 300 000 km^2^ (Fuchs et al. 2013). Since 1990, the annual forest area increase has averaged 0.3%, with the highest rates being found in South-West Europe (+ 0.78%) and South-East Europe (+ 0.38%) (Forest Europe 2020). Increasing forest areas has been favoured by European and national policies for a long time through subsidized active forest restoration under the Common Agriculture Policy (CAP). However, a significant share of these new forests were not planted but are the result of natural forest expansion (NFE), that is, the expansion of secondary forest through natural succession on non-forest land (thus implying a land cover change) (FAO 2020). NFE is typically an ‘unintended’ process caused by a variety of socio-economic, political and environmental factors, often relating to a lack of profitable alternative land use practices resulting in land abandonment (Rey Benayas 2007).

This phenomenon is likely to continue in the coming decades; a recent study estimates that no less than 200 000 km^2^ of EU farmlands are under high probability of abandonment between 2015 and 2030 (Perpiña Castillo et al. 2018). Although its contribution to the forest area increase across Europe is very difficult to quantify precisely, diverse regional-scale estimates imply that secondary forests formed by NFE cover today at least several tens of thousands of km^2^ (e.g. Schierhorn et al. 2013; Potapov et al. 2015; Buitenwerf et al. 2018; Palmero-Iniesta et al. 2021). Studies suggest that 2/3 of the forest on agricultural land in the EU has regenerated naturally (Perpiña Castillo et al. 2018).

Forests play a central role in several major EU policy initiatives, owing to their critical importance for addressing the twin crises of climate change and biodiversity loss. The European Green Deal considers forests crucial for mitigating climate change, particularly through carbon sequestration from the atmosphere (European Commission 2019). Forests are also a critical subject of climate change adaptation and play a key role in meeting targets under the EU Biodiversity Strategy 2030. The Biodiversity Strategy proposes a EU Nature Restoration Plan “to increase the quantity, quality and resilience of its forests” (European Commission 2020, p. 10). One target set by both the Biodiversity Strategy and the EU Forest Strategy 2030 is to plant 3 thousand million additional trees in the EU by 2030 “in full respect of ecological principles”, and to secure the trees “for several decades” to increase the forest area by 2000–3000 km^2^ per year in addition to the current forest area projections that include NFE (European Commission 2022b, pp. 4 and 7). Thus, NFE is not considered as an instrument to achieve the additional 3 thousand million tree target but is implicitly accounted for under the business-as-usual scenario. The EU Forest Strategy does explicitly mention the significant role of NFE: “Spontaneous forest regrowth through natural succession is the main force driving the increase of forested areas in the EU, mostly associated with abandonment of agriculture and rural areas” (European Commission 2021, p. 15). Although no further details or guidance on NFE is given, it acknowledges the potential of NFE for a forest restoration policy agenda; to our knowledge, this is the first such acknowledgement in a EU policy document. Furthermore, the European Commission launched a proposal for a EU Nature Restoration Regulation in June 2022, which foresees restoration beyond the Natura 2000 Network habitats; if adapted as currently suggested, this would include NFE on abandoned land (European Commission 2022a).

Recent interdisciplinary research underlines the potential of NFE for creating multifunctional, self-sustaining ecosystems that can provide diverse ecosystem services (Cruz‐Alonso et al. 2019; Chazdon et al. 2020; Martín‐Forés et al. 2020). However, research also shows the potential risks of NFE—for instance, related to a loss of cultural open landscapes (MacDonald et al. 2000; Plieninger et al. 2014) or to wildfires (Ursino and Romano 2014). A systematic assessment of the potential of NFE to contribute to European forest restoration is lacking. This paper provides such an assessment, based on existing literature in relevant research disciplines. Specifically, we have been screening the relevant European literature on the phenomena from a variety of relevant disciplines, including ecology and forest management, climate science, sociology, political science, and economics, and explore based on that the main challenges and opportunities relating to NFE from different angles. Subsequently, we outline recommendations for policymakers to unfold opportunities and to deal with existing challenges regarding NFE.

Specifically, we ask:

- What is known about the challenges and opportunities connected to NFE in relation to the EU’s forest policy objectives?
- What can be concluded for the policymaking on NFE in Europe?

## Challenges and opportunities of NFE

### Biodiversity

The establishment of secondary forests resulting from NFE (from now on ‘secondary forests’, if not stated differently) and associated succession processes generate a consistent increase in the area, biomass, vegetation structural complexity and species richness of woody habitats. New forests are typically colonised very quickly by common, mobile and generalist species (Espelta et al. 2020; Prach and Pyšek 2001; Whytock et al. 2018; Valdés-Correcher et al. 2019), especially when they are well connected to source habitats in the surrounding landscape matrix (Cruz-Alonso et al. 2021). Hence, secondary forests can quickly exhibit levels of taxonomic and functional diversity comparable to those observed in long-existing forests sharing the same structural characteristics (Espelta et al. 2020). However, the arrival of regionally rare, not very mobile and specialist species and the associated build-up of complex multi-species networks of biotic interactions can require many decades or centuries (Jacquemyn et al. 2001; De Frenne et al. 2011; Correia et al. 2021). Hence, even extensive secondary forests cannot compensate for the loss of old-growth forests with their unique biodiversity (including many highly specialized species), structure and functioning (Selva et al. 2020).

From a biodiversity conservation perspective, NFE can have a variety of positive and negative effects. NFE has significantly contributed to forest connectivity and defragmentation across Europe (Palmero-Iniesta et al. 2020). This process has favoured numerous forest-dwelling species including birds (Whytock et al. 2018), Lepidoptera (Ruiz‐Carbayo et al. 2017) and Diptera (Fuller et al. 2018). New secondary forests can also serve as habitats and ‘stepping stones’ for the expansion of invasive species (With 2002). Moreover, NFE represents a major challenge for the conservation management of species-rich, semi-natural open habitats formed by historical extensive livestock farming (WallisDeVries et al. 2002; Calaciura and Spinelli 2008), causing a rarefaction and local extinction of species living in such habitats, including butterflies, birds and plants (Plieninger et al. 2013; Melero et al. 2016; Regos et al. 2016). Nevertheless, NFE is not a primary driver of the widespread decrease of habitat diversity (i.e. landscape homogenisation) (Palmero-Iniesta et al. 2020), a trend mostly caused by agricultural intensification.

Overall, the effects of NFE on biodiversity and its conservation are highly context-specific. They usually depend on components such as (i) the type of habitats that new forests are replacing (e.g. arable lands, industrial wastelands, species-rich grasslands), (ii) the surrounding landscape matrix and its species pool (e.g. forest area, productivity, fragmentation level), (iii) the extent and spatial distribution of NFE processes (e.g. colonisation of little spots in the landscape vs. large continuous areas), and (iv) the time elapsed since the abandonment of former land uses. As a consequence, the challenge for landscape and conservation management consists in ensuring that the potential effects of NFE on biodiversity are addressed at a proper spatial (i.e. local and landscape) and temporal (i.e. long-term) scale (Whytock et al. 2018), weighing associated benefits and trade-offs in relation with other land uses.

### Climate change mitigation

NFE bears extensive opportunities for climate change mitigation through carbon sequestration and regulation (Navarro and Pereira 2012). Regional and global studies have highlighted the great potential of regrowing secondary forests (planted or naturally grown) to act as carbon sinks (Vilà-Cabrera et al. 2017; Cook-Patton et al. 2020). The carbon sequestration potential of NFE is not merely an effect of increasing forest area but is also linked to some particularities of trees growing on former croplands and pastures, mostly related to physicochemical soil legacies. Firstly, past agricultural land use often results in soils with higher nitrogen and phosphorus content (Compton and Boone 2000; Fraterrigo et al. 2005), which tends to enhance tree growth (Alfaro-Sánchez et al. 2019) and boost above-ground biomass productivity (Poorter et al. 2016). Secondly, former agricultural soils tend to be deeper but poorer in soil organic carbon than soils with long-existing forests (Clark and Johnson 2011; Wertebach et al. 2017). This provides the opportunity of storing a considerably larger amount of carbon in agricultural soils than in more saturated forest soils. Ultimately, as long as wildfire risk is managed, NFE growth would offset a significant amount of carbon emitted (e.g. 9% of the total emissions in Spain between 1986 and 2007; Vilà-Cabrera et al. 2017).

Notwithstanding the above-mentioned benefits, the future potential of NFE for climate change mitigation in the EU is subject to some challenges concerning: (i) a certain mismatch between areas of highest carbon sequestration potential and areas where land abandonment occurs (see Cook-Patton et al. 2020), and (ii) the resilience of secondary forests to climate change related disturbances. Although extensive farmland surfaces are projected to be abandoned in the EU by 2030 (Perpiña Castillo et al. 2018), this trend is predicted to occur mostly in areas with restricted plant growth potential (this being one of the reasons for agriculture cessation). This is the case for the Mediterranean region, where tree growth associated with NFE may benefit less from the biological and physicochemical legacies of abandoned agricultural soils owing to climatic constraints (Palmero-Iniesta et al. 2021), therefore limiting the mitigation potential of NFE. In addition, the higher growth rates observed in secondary forests in comparison to long-established ones may also come with increasing disturbance risks constraining the potential for climate change mitigation. This is the case if growth occurs at the expense of changes in functional traits (e.g. leaf area index, wood density, root morphology) that control tree resilience to disturbances (e.g. drought, insect pests, wildfires, storms). In line with this, Mausolf et al. (2018) observed that naturally regrown beech forests on former agricultural lands in Germany exhibited a greater growth reduction during adverse climatic conditions compared to long-existing forests, probably owing to the smaller root systems they developed in more fertile soils. Similarly, Alfaro-Sánchez et al. (2019, 2021) reported lower wood density and an overall higher sensitivity to climate-induced stress in naturally regrown forests in Spain. Besides functional attributes, the species composition of naturally regrown new forests may also condition their response to disturbances. For instance, these forests have exhibited more resistance to insect herbivory than long-existing forests (Espelta et al. 2020; Ruiz-Carbayo et al. 2020); yet they exhibited a lower resistance and regeneration ability after wildfire (Puerta-Piñero et al. 2012).

Summing up, NFE definitely holds significant potential for climate change mitigation in Europe and elsewhere. Risks from climate change and related disturbances need to be accounted for and specific management measures may be needed to increase the resilience of naturally regrown forests to such risks, particularly in Southern Europe.

### Climate change adaptation

NFE can support climate change adaptation at two different scales: (i) ecological and evolutionary processes can help secondary forests to increase their own resilience, and ultimately persistence, in a changing environment; and (ii) secondary forests can contribute to the adaptation of wooded landscapes as a whole. For the first, naturally regrown forests tend to exhibit structural and ecophysiological characteristics that may confer on them a different resilience to climate change and associated disturbances (e.g. windstorms, drought, insect pests) compared to both tree plantations or long-existing forests. As for the comparison with tree plantations, the resistance of secondary forests to windthrow benefits from a heterogeneous canopy structure generated by the successive and irregular tree recruitment that characterises them. Compared to long-existing forests, the newly established forests benefit from a tendency of trees growing under high levels of solar radiation to invest more resources in radial increment and less in height growth which increases their resilience (Mitchell 2013). The tree recruitment under high solar radiation in secondary forests could also explain observations that trees from such forests tend to display a higher water use efficiency than those from long-existing stands, acquired through the development of a lower specific leaf area (Acuña-Míguez et al. 2020; Guerrieri et al. 2021). On the other hand, trees resulting from NFE often tend to grow faster and to develop lower-density wood compared to long-existing forests, which potentially increases their susceptibility to drought stress (Alfaro-Sánchez et al. 2019; but see Espelta et al. 2020). Future studies have to elucidate which of the involved ecological and ecophysiological mechanisms will be determinants for the resilience of secondary forests to increasing drought and windthrow risks. In any case, extensive tree mortality following climatic extreme events tends to enhance the natural recruitment of young trees and to favour rapid vegetation recovery (Lloret et al. 2012), unless it occurs over large areas. From a long-term perspective, such enhanced recruitment can favour the spread of drought-resistant genotypes and ultimately the microevolutionary adaptation of such forests to novel climatic conditions, an effect that can only be observed in forests that regrow naturally (Petit and Hampe 2006; Saleh et al. 2022).

Secondary forests show not only extensive variation in tree height and density, but also a diverse composition (Basnou et al. 2016) and sometimes higher diversity of woody plant species than planted forests (Cruz‐Alonso et al. 2019) or long-existing managed forests (Espelta et al. 2020). A higher number of tree species provides ecological insurance against different disturbances; increasing tree species diversity is considered one of the pillars in helping forest ecosystems cope with environmental disturbances (Jactel et al. 2017). In the particular case of insect pests, mixed-species forests resulting from NFE probably benefit from a low appearance of host trees for insect herbivores (Castagneyrol et al. 2013) as well as from a high variation in plant palatability, which helps reduce herbivore performance (Wetzel et al. 2016). Future studies have to address the relevance of this effect during pest outbreaks to better understand the resilience of secondary forests resulting from NFE to this particular type of climate change impact.

On the landscape scale, NFE may help create more resilient forest landscapes by contributing to the development of functional complex networks (sensu Messier et al. 2019) of forest patches varying in tree species composition. As tree species composition of secondary forests patches stemming from NFE is more different among them than other types of forests (Espelta et al. 2020; Cruz-Alonso et al. 2021), they may serve as reservoirs for many woody plant species as well as other favourable biotic agents, which can then colonise surrounding forests. In cases of high wildfire risk, however, NFE—same as planted forests—may contribute to fuel networks; these negative aspects may require specific management measures. Further research is needed to provide empirical evidence of the role of NFE for the local adaptation of forests and wooded landscapes to climate change across different local contexts.

### Forestry and economic use

NFE is increasing forest biomass substantially in some regions. This brings a potential for additional forest biomass use for forestry and a forest-based circular economy. Beyond woody biomass, the new forests can provide non-wood forest products, as well as opportunities for other ecosystem services that can be economically valuable to local communities.

The wood usage potential of NFE depends on various factors. The forest composition and structure are important; for instance, some of the colonising species might not be marketable or they might be protected by law, such as some *Juniperus* spp. in Spain. The most immediate use for recently grown trees is bioenergy (fuelwood, chips, pellets), as small diameter trees of almost any species can be used. While reactivating these lands for fuelwood production may be profitable only under certain accessibility and machinery circumstances (Elyakime et al. 2011), fuelwood usage is attractive as it has a relatively short rotation and a rather good market (Piussi and Pettenella 2000). Medium-sized diameters (> 25 cm) of coniferous may well serve the demands of the pallet and cross-laminated timber industry—the latter having a considerably higher added value than the former. Yet, the forest industry is probably not present in many regions where NFE occurs to a large extent, although the forest industry is expected to expand in some regions related to the promotion of sustainable wood construction (Fraser 2017; Jonsson et al. 2021). Furthermore, the potential usage depends on the legal provisions restricting biomass harvesting, such as the administrative difficulty of changing the registered land use category from agriculture to forest land, or of fulfilling the requirements for forest management plans and harvest permits (Nichiforel et al. 2018). Additionally, the potential of NFE can depend on technological harvesting limitations as well as on the existing value chains in the demand area and on broader socioeconomic factors determining economic feasibility of forest management.

As well as firewood, NFE can provide a diversity of non-timber products such as fungi, fruits, herbs and game. The new forests can also provide shelter to various organisms, which might result in positive externalities for surrounding crops (e.g. pollinators, predators of agricultural pests; Rey Benayas and Bullock 2012), or in disservices (e.g. wild boar, roe deer). For example, fungi of economic interest will appear spontaneously if the mycelium spreads along with tree colonisation. Previous cereal parcels and successional shrublands are well suited to host (black) truffle mycorrhized oaks (Reyna Doménech et al. 2002; Taschen et al. 2015). Pine-dominated areas are adequate hosts of symbiotic mushroom species that are in high demand (de Aragón et al. 2007). Valuable edible nuts start developing relatively early—at stands of approximately 10 years for conifers (e.g. *Pinus*) and 20 years for broadleaved species (e.g. *Quercus, Castanea*). Some aromatic (e.g. *Thymus, Rosmarinus*), cosmetic (e.g. *Cistus ladanifer*) and medicinal (e.g. *Arctostaphylos avaursi*, *Glycyrrhiza glabra*) plants may be the first to colonise the new forests (Cristóbal et al. 2020). Pine resin can be harvested once the trees have reached a threshold diameter, which takes 50 years (Pinillos et al. 2009). Cork can be commercially harvested from cork oak (*Quercus suber*) once they are 20–30 years old. Furthermore, NFE offers possibilities of gathering forest materials for decorative uses, such as pinecones or heather (Lovrić et al. 2020). Artisanal handcraft is also possible from shrubs colonising these NFE (e.g. *Buxus sempervirens*, *Salix fragilis*). Additionally, secondary forests can harbour animal-related economic activities, such as honey production, hunting or silvopastoralism (Gortázar et al. 2000).

Next to providing economic opportunities, related forest management interventions such as tree harvesting, pruning, species diversification and grazing introduction may provide co-benefits, such as reducing the fuel ladder structures to lower the risk of canopy fire, reducing tree density to increase water yields, or increasing human accessibility to improve recreational use. However, the socioeconomic factors that have triggered (agricultural) land abandonment will possibly hamper forest use options. This includes accessibility for mechanised harvesting, stand productivity, labour availability and regional demand for products (Frei et al. 2020). It remains an open question how far technological innovations (e.g. increasing harvesting robotization; Parker et al. 2016), will increase profitable forest use options, with future machinery potentially reaching previously inaccessible areas.

### Societal perceptions

The transition of former agricultural land into forest is a significant land use change impacting people across Europe. There is a need to assess and consider the perspectives, needs and interests of those owning and potentially working with the land, as well as the wider network of related societal groups, including visitors such as recreationists.

Societal perceptions related to land abandonment and NFE have been studied in different countries in Europe. These studies mostly focus on the early stages of NFE after land has been abandoned. While findings are clearly context-dependent, there are some shared patterns that can be made out at the local scale. Studies reveal opportunities related to environmental, forest, rural development and tourism, as actors consider benefits through new ecosystem services provided by NFE in the future. These are partly connected to the development of wilderness through naturally evolving ecosystems (Höchtl et al. 2005; Frei et al. 2020), to recreational opportunities especially when NFE occurs close to urban areas (Martín‐Forés et al. 2020), as well as a potential increase in forest biodiversity and forest-related goods.

However, results largely show that local actors involved in land management (e.g. farmers, landowners) often have negative and defensive attitudes towards agricultural land abandonment and NFE. The main reasons for such negative perceptions are connected to the loss of cultural landscapes—long characterized by agricultural practices, often intertwined with local culture and traditions—and the related socio-economic consequences for (rural) livelihoods (Soliva et al. 2008; Frei et al. 2020). This adds ‘emotional and cultural dimensions of change’ to NFE (Fernández-Giménez 2015, p. 1). Groups attached to these former land use practices prefer cultivated landscapes, characterized by traditional agricultural mosaics, such as silvopastoral systems in the Mediterranean climate region. From an aesthetic viewpoint, which also plays a role for tourism, the traditional landscapes stand in contrast to unmanaged forests emerging from NFE; if land transition occurs on a large scale, this can affect the scenery (e.g. Bieling 2013). The attachment to cultivated landscapes with a mixture of open and forested land has been documented for many European regions (see for instance Soliva et al. 2008; Bieling 2013; Ruskule et al. 2013; van der Zanden et al. 2018; Zagaria et al. 2018). Furthermore, the initial stages of NFE and a lack of management tends to be problematized by land use managers and owners, connected to the perceived need for ‘regular’ forest management (Frei et al. 2020). In relation to this, new forests are associated with increased risks, such as forest wildfires (Soliva et al. 2008; Frei et al. 2020).

Conflicting perceptions relate to different socioeconomic interests, ways of life and worldviews, connected to farming, forestry, recreation or conservation (Soliva and Hunziker 2009; Martín‐Forés et al. 2020). Additionally, the generational, educational and geographical context can play a role; the younger generation or urban actors may value the nature and leisure aspect of NFE more than others (Ruskule et al. 2013; Martín‐Forés et al. 2020; Zoderer and Tasser 2021). This indicates some potential conflict regarding the spatial distribution of NFE: while if often (although not only) occurs in sparsely populated regions with marginal lands, the strongest demand for recreational landscapes and forests as green spaces occurs in peri-urban areas (Frei et al. 2020; Barnaud et al. 2021).

In summary, there is a need to balance expectations and demands originating from different actors and scales of policymaking, particularly between the local and European levels. Adequate management options for NFE need to be based on the local contexts.

### Policymaking

NFE has now been recognized in the EU Forest Strategy as an important driver of forest area increase and may play a bigger role under a new EU restoration law. Yet it is still not explicitly addressed in most EU policies. An implicit focus on the phenomenon is connected to land abandonment in agriculture and rural development policy. Here, NFE has mainly been considered from the viewpoint of avoiding agricultural land abandonment; CAP measures have aimed to keep the agricultural system running, including agricultural re-use with respective measures under the CAP (Varela et al. 2020; Fayet et al. 2022), while at the same time active reforestation was supported. This political neglect of NFE is remarkable as the process offers cost-effective opportunities from a policy perspective. Since NFE occurs naturally, no budget, resources, people nor programmes are needed for forests to grow, making it less costly than active restoration measures.

Investigating the existing literature, some challenges become apparent that help to explain why NFE has been neglected as a policy issue at the European scale. NFE on abandoned agricultural land is a topic that spans different policy sectors with diverging interests and perspectives on the issue, above all agriculture, forestry and conservation (Varela et al. 2020; Frei et al. 2022). Different policy objectives for forests in these sectors and a lack of policy integration at the EU level (Winkel and Sotirov 2016; Sotirov et al. 2021) make it challenging to take coordinated policy action regarding NFE (Varela et al. 2020; Frei et al. 2022).

Furthermore, NFE is an ecological process that occurs without any need of active policymaking. This may go against the usual bureaucratic and sectoral interests, which favour ‘active’ policymaking and giving mandates and resources to public agencies (Krott 2005). Active processes such as afforestation or subsidizing agricultural use align better with this logic; passive ecological processes may be considered less politically ‘capable’. Additionally, potentially useful management trajectories are highly context-dependent, related to, for instance, the ecological, socioeconomic and/or land-tenure situation (Frei et al. 2020). These aspects may make NFE less suitable for policymaking at higher (EU) levels.

Lastly, there is a lack of political will to act on NFE. There are only a few policy actors with an explicit interest in NFE (Fayet et al. 2022; Frei et al. 2022). In a study in France and Spain, NFE was shown to be incompatible with traditional policy narratives of the affected policy sectors. Conservation actors tend to focus on old-growth forests with their specific biodiversity, or on traditional, extensively used mosaic landscapes, which are seen as being threatened by NFE. Forestry actors focus on the management of existing forests and plantations rather than on the comparatively young successional forests, which are of only limited economic interest in the early stage of NFE. Agricultural actors tend to focus either on agricultural boom regions, where NFE does not occur, or they see NFE as a process to be stopped or even reversed by subsidizing agriculture (Frei et al. 2022). For a few years now, some non-governmental actors such as the NGO Rewilding Europe have been actively promoting rewilding and wildlife comeback on abandoned land, highlighting its benefits through a “nature-based economy” (Rewilding Europe 2022), a concept that can also be found in the academic literature (Bassi et al. 2022). Thus, new narratives connected to land abandonment may be emerging (Frei et al. 2022). Although still largely missing at a European scale, some studies have found specific policymaking connected to NFE at the local level, directly connected to management and land use planning (e.g. in Scotland, see Barnaud et al. 2021).

In sum, the potential of NFE to contribute to restoration policy objectives has, at least in the past, hardly been considered in European level policymaking. This may change slightly in the near future due to a stronger focus on forest restoration at the EU level, but policy-related challenges continue to exist.

### Summary

Table 1 summarizes the main challenges and opportunities of NFE for each topic discussed.

**Table 1:** Overview of the main challenges and opportunities of NFE

Topic	Opportunities	Challenges
Biodiversity	**•** Increase in forest habitat area, structural complexity and species richness, especially in the long-term **•** Increase of landscape heterogeneity, forest connectivity and defragmentation depending on the distribution of forest regrowth across the landscape (mosaic structure) **•** Habitats for agricultural auxiliars (e.g. pollinators, predators of agricultural pests)	**•** Rarefaction and local extinction of open landscapes and species depending on them **•** Habitats and connectivity may also favour invasive species
Climate change mitigation	**•** Effective carbon sequestration connected to carbon accumulation potential of young forests **•** Additional carbon mitigation potential rooted in agricultural soil legacies that can lead to enhanced tree growth and carbon capture	**•** Areas of highest carbon sequestration potential not matching areas with land abandonment at present **•** Increasing risk of climate change disturbances may negatively affect long-term mitigation potential of forests
Climate change adaptation	**•** Naturally regenerated stands with heterogeneous structure increasing resistance and resilience to disturbances **•** NFE growth conditions favouring acclimation and selection for drought resistance **•** Increase in functional diversity supporting resilience of new forests and of the whole wooded landscapes	**•** NFE species composition constrained by local resources **•** In some situations, NFE requires specific risk management measures (e.g. wildfire)
Forestry and economic use	**•** Wood usage potential, depending on forest composition, management and socio-economic feasibility **•** Provision of non-wood forest products, such as mushrooms, nuts, and resin **•** Supply of other (non-provisioning) ecosystem services, such as accessibility for recreation or erosion control	**•** Context-specific socioeconomic factors preventing forest use, such as labour availability, regional demand for products, accessibility for mechanisation, and productivity **•** NFE providing habitat to species that cause damages in surrounding agricultural areas, such as wild boar, roe deer or wild goats
Societal perceptions	**•** NFE providing new land use options, for instance, to tourism, recreation and forest-related goods, potentially supporting a positive attitude towards NFE **•** Positive attitude towards NFE as wilderness and recreational area	**•** Negative attitude towards NFE scenery, as a symbol of the decline of rural livelihood and the loss of cultural landscapes and aesthetic values **•** Conflicting perceptions related to different socioeconomic interests
Policymaking	**•** Naturally occurring restoration of forest and forest area increase, supporting respective EU and national policy objectives **•** Cost-effective process taking place without additional funding needed depending on future land use objectives	**•** NFE as intersectoral topic leading to conflicts where sectors have fundamentally different objectives for these lands **•** Neglect of NFE at EU policymaking level and currently a lack of specific policy strategies regarding NFE as a tool for restoration

## Policy recommendations

### Integrate NFE as a tool for European forest restoration policy

As shown above, NFE can contribute significantly to the objectives of European land use, forest and environmental policies. So far, EU policies have hardly explored this potential, mostly ignoring the process. Hence, a first recommendation is to explicitly consider NFE as an important process of forest restoration and to develop explicit policies to support and manage the process. The current discussion about EU-level restoration legislation includes ideas about requesting Member States to develop national restoration plans and considering habitats beyond the Natura 2000 Network. This discussion could be a good starting point for explicitly addressing NFE.

Nevertheless, NFE is not a silver bullet. While the process has happened and is happening at significant scales, an active consideration of different management and conservation options may be needed to best exploit its potential for nature and society, at least under past and current socioeconomic conditions of land management. As the above discussion has shown, governance and management concepts need to be connected to:

- The respective main objectives for the new forests, spanning climate change mitigation and adaptation, biodiversity conservation, as well as different types of forest use;
- The socioeconomic settings in which NFE occurs that enable or constrain management options;
- The societal interests and perceptions towards these forests that enable or prevent different management approaches.

This paper illustrates that NFE can be evaluated quite differently. Thus, NFE needs to be considered from different angles, not only from the perspective of climate change mitigation and biodiversity conservation, but also considering different forestry uses and the socioeconomic contributions of forests to rural development, ranging from woody biomass to non-wood forest products and multiple locally valuable forest ecosystem services. Involving different sectoral and societal views calls for policy integration; this requires processes to integrate different concerns in conservation and management planning, and necessitates addressing trade-offs. Finally, and possibly most importantly, the highly imbalanced geographical distribution of NFE poses a significant challenge. It rarely occurs in fertile landscapes characterized by intense agricultural use, nor areas with little forest area—i.e. the areas where natural reforestation could bring the highest benefits from a biodiversity or recreational perspective. Along with making better use of an ongoing process caused by changing socioeconomic conditions in the periphery of Europe’s agriculture, NFE could be actively encouraged in regions where it will not occur without intervention but where it may have the highest benefits for biodiversity and people*.* These latter regions require consideration of the likely much higher costs and trade-offs with agricultural production or infrastructural development.

### Develop regional strategies that place restoration management into the context of local needs

There are different options for managing NFE on abandoned land. First, abandoned land can be afforested to create new forests. Second, abandonment can be tackled and reverted resulting in a re-use of agriculture—most likely with extensive agriculture, but intensification is also possible. To keep extensive agriculture running, or to revert to it, requires finding a sustainable socioeconomic basis, for instance in combination with tourism and landscape subsidies (Varela et al. 2020). Lastly, NFE is often the ‘natural trajectory’ on abandoned land, making it potentially useful for forestry; it may also be useful for non/low-intervention conservation approach, by implementing active or passive rewilding.

Deciding where a given scenario can occur and if it is suitable requires support from policymaking and land planning. As precondition for any of the mentioned management options, regional inclusive governance processes are needed to identify concepts for how to manage NFE, including the option of non-intervention approaches. Restoration objectives may determine the value and potential of NFE. Local needs and visions need to be balanced against national and bigger European policy objectives. Different perceptions and land use ‘ideologies’ and interests connected to NFE need to be kept in mind; space should be given to elaborate multiple viewpoints so as to develop shared land use scenarios. Trade-offs are necessarily part of decisions about what direction to take, at least at the local scale. This calls for regional restoration assessments reflecting on the potential of NFE as a tool to reach restoration goals. If the EU implements a restoration legislation in the future, NFE can and should be included as one tool to increase forest area. NFE can support some of the forest restoration indicators that were discussed in a recent proposal by the Commission, namely forest connectivity, common forest bird index and organic carbon stock (European Commission 2022a).

### Support interdisciplinary research and monitoring on NFE

Our assessment has demonstrated the importance of considering multiple perspectives in the assessment of NFE; hence, more interdisciplinary research is needed to explore different facets of NFE comprehensively. Improved knowledge and data are required to answer important questions at the European scale, about where NFE occurs and in what contexts, including the elaboration of future development trajectories. This needs to involve both ecological and socioeconomic dimensions (Barnaud et al. 2021; Frei et al. 2022). On the natural science side, a better understanding of the quantity and distribution of NFE is key, but also the ‘quality’ of NFE (i.e. analysing the composition and dynamics of the new forests). Regarding biodiversity, while there is rich data for habitats under the Natura 2000 Network, sound data for habitats beyond the network is often missing and is much needed (Costa Domingo et al. 2022). From a social science perspective, there is a need to better understand the existing policymaking and broader governance scheme of NFE at regional/local levels, and how suitable policy strategies could act as role model for the national restoration plans as required under the Nature Restoration Regulation. In any case, research on NFE should enable opening up perspectives about potential risks and benefits, without being overly supportive for only one trajectory of land abandonment, as has often happened in the past (Dolton-Thornton 2021).

## Conclusions

This paper assesses the challenges and opportunities of NFE for the current forest restoration agenda in Europe. Specifically, we discuss NFE against the background of existing research connected to biodiversity, climate change adaptation and mitigation, forestry and economic use, societal perceptions and policymaking. Thereby, we find opportunities and challenges connected to NFE as a forest type and as a new forest area on former agricultural land. We argue that opportunities connected to NFE exist, if the ecological and socioeconomic context allows and if respective management measures are taken up to deal with trade-offs and associated risks. Up to now, however, NFE has hardly been considered as a tool for restoration at European scale. We suggest taking NFE into account as a tool under EU restoration policies and beyond, while not losing sight of associated challenges and trade-offs with other policy objectives.
